# Physiotherapeutic care of patients with rupture of the anterior cruciate ligament or meniscus lesion in Germany

**DOI:** 10.1007/s43999-026-00091-7

**Published:** 2026-10-06

**Authors:** Anna Gurok, Andreas Voss

**Affiliations:** 1 Department of Trauma, Hand, Plastic, and Reconstructive Surgery, Julius- Maximilians-University of Wuerzburg, Wuerzburg, Germany; 2 Sporthopaedicum Regensburg, Regensburg, Germany; 3 Department of Trauma Surgery, University Hospital Regensburg, Franz-Josef-Strauß-Allee 11, 93053 Regensburg, Germany

**Keywords:** Physiotherapy prescription, ACL rehabilitation, Meniscus lesion rehabilitation, German catalogue of treatments

## Abstract

**Introduction:**

Physiotherapy plays a central role in the rehabilitation of musculoskeletal injuries. While the German catalogue of therapies regulates the type and frequency of treatments in Germany, there is a lack of data on actual prescription practice. The research question of this survey was to examine the current physiotherapeutic care and prescription practices for patients with a rupture of the anterior cruciate ligament (ACL) or a meniscus lesion.

**Methodology:**

An online questionnaire was developed and sent to practitioners who regularly treat patients with knee joint injuries. They were asked about current care and the usual prescription figures for treatments as well as the availability of physiotherapy care.

**Results:**

Between May 28 and June 30, 2024, n = 156 participants completed the survey. Therapeutic exercises are the most commonly prescribed treatment for ACL ruptures and meniscus lesions in the first six months after surgery (prescribed by over 97% of practitioners). On average, 27.1 sessions (SD = 13.6) of therapeutic exercise were prescribed in the first year after an ACL rupture. On average, 14.9 sessions (SD = 10) of therapeutic exercise were prescribed in the first year after a meniscus lesion. More than half of respondents reported waiting times for physiotherapy of over two weeks.

**Conclusion:**

Therapeutic exercises dominate as a form of prescription and are already prescribed in isolation in greater numbers than the guideline treatment values in the German catalogue of therapies after ACL ruptures. Long waiting times and limited availability pose challenges to ensuring that the prescribed sessions can be used.

## Introduction

Physiotherapeutic care in Germany is an essential component of the healthcare system and plays a crucial role in the prevention, treatment, and rehabilitation of musculoskeletal disorders. According to data from the Robert Koch Institute, 25.5% of women and 17.7% of men made use of physiotherapeutic services within a year [1].

Knee joint injuries place a burden on the healthcare system due to their incidence and the duration of post-treatment. The most common knee joint injuries include anterior cruciate ligament (ACL) injuries and meniscus lesions [2]. Physiotherapy is a central component of treatment, both in conservative management and perioperative care. Ardern et al. find that while 81% of patients return to sports after surgical reconstruction of the ACL, only 65% return to their pre-injury level [3]. Furthermore, the risk of re-rupture or contralateral injury of the ACL is increased after reconstruction [4, 5]. In addition to young age and high activity levels, altered neuromuscular function and biomechanics are considered key risk factors [4, 5]. In young athletes, the risk of a repeat ACL injury after reconstruction is even estimated to be increased 30 to 40 times [6]. Effective treatment of patients with ACL injuries or meniscus lesions is of tremendous relevance not only in the short to medium term; proper care is essential in the long term as well. The risk of developing osteoarthritis is increased 4 to 6-fold after these injuries, highlighting the need for adequate physiotherapeutic aftercare [7]. Following ACL reconstruction, rehabilitation is typically structured as a phased, criteria-based progression spanning several months. A return to low-impact sports is generally considered at approximately 4–6 months, whereas high-impact and pivoting activities are typically restricted until 9–12 months post-surgery, ideally guided by functional testing and orthopedic evaluation [8, 9]. In contrast, rehabilitation following meniscal surgery depends on the specific procedure. Partial meniscectomy allows for accelerated recovery, with early full weight-bearing and a return to sport often achieved within 4–12 weeks. Conversely, meniscal repair necessitates a more conservative, protective approach to ensure tissue healing; consequently, return to sport is typically delayed until at least 4 months, extending to 9 months in complex cases [8, 10]. Overall, ACL rehabilitation is more prolonged and highly structured compared to meniscal procedures [11].

The German catalogue of therapies serves as a reference for prescribing therapeutic sessions, particularly in physiotherapy, and is an integral component of the Therapeutic Treatment Guideline [12].

Within the German catalogue of therapies, specific diagnoses are grouped and prescribable treatments as well as recommendations about the type, number, and frequency of treatments are specified. In this process, the physician selects from different physiotherapy modalities, each targeting specific leading symptoms and therapeutic objectives. While therapists can provide reports that might inform future prescriptions, their input is seldom taken into consideration in actual practice [13]. In the diagnostic group of diseases of the extremities and the pelvis, as a rule, 6 sessions per prescription can be prescribed, the orienting treatment quantity amounts to up to 18 sessions. If, beyond that, physiotherapy is still needed in the individual case, treatments can be prescribed ‘for medical reasons’.

In the German clinical guidelines for the treatment of ACL injuries and meniscal lesions, physiotherapeutic measures for both postoperative rehabilitation and conservative management are firmly established [14, 15]. Important goals of physiotherapeutic treatment for these injuries include restoring adequate mobility and proprioception through therapeutic exercise, strengthening the musculature and improving neuromuscular control using device-based exercises, resolving joint blockages and improving joint function through mobilizing and manipulative manual therapy techniques that also modulate nociception, and addressing oedema and tissue decongestion in patients with swelling using manual lymphatic drainage [9, 16, 17]. In contrast, physiotherapy faces several challenges: the capacity of physiotherapy practices is limited by factors such as a shortage of qualified specialists. This can lead to longer waiting times for patients, and if surgery dates and therapy appointments are not properly coordinated, gaps in care may occur. Yet rehabilitation measures should begin as early as possible, as delays can slow the healing process and potentially result in poorer postoperative outcomes [9].

Nolte et al. show that, in practical implementation, prescriptions predominantly consist of basic therapeutic exercises and manual therapy as the primary modalities [13]. Currently, there is a lack of data regarding actual physiotherapeutic care and prescription practices for patients following an ACL rupture or a meniscus lesion. Therefore, this study conducted a survey among physicians who regularly treat these injuries to investigate the current prescription situation for these indications. The primary aim was to examine the actual physiotherapeutic care and prescription practices and to understand how structural constraints shape clinical decision-making. Furthermore, by analyzing real-world patterns, this study seeks to identify potential discrepancies between guideline recommendations, what physicians consider an adquate amount of therapy, and their actual routine practice.

## Methods

### Questionnaire

A questionnaire with various sections was created using the web application SoSci Survey (SoSci Survey GmbH, Germany). The content was newly developed in consultation with board members of the professional associations (details in participant acquisition section). In the first part, general information was collected regarding the therapy focus, the patients, and the number of cases treated by the participating physicians for meniscus lesions or ACL ruptures. In the second section, participants were asked about their usual prescription volume of physiotherapy sessions for legally insured patients with a meniscus lesion or ACL rupture. A distinction was made between the different types of prescribable treatments. In addition to the actual prescription practice, the participants were asked to provide information about the number of prescriptions they considered adequate. The last section included further questions about the physiotherapeutic pre- and postoperative treatment of the patients. Pre-testing within an internal committee was conducted to assess comprehension and ensure usability. The complete questionnaire can be found in the appendix. The research project and study protocol were submitted to the Ethics Committee of the University Hospital Regensburg. It was confirmed that the aforementioned study does not require professional advice according to Sect.  15 of the Professional Code of Conduct for Doctors in Bavaria and therefore no vote by the Ethics Committee is required.

### Participant acquisition

The online questionnaires were sent to physicians via the e-mail distribution lists of the professional associations of Arbeitsgemeinschaft Arthroskopie (AGA), Berufsverbands für Arthroskopie e. V. (BVASK) and Berufsverbands für Orthopädie und Unfallchirurgie e.V. (BVOU). Participation in the survey was voluntary and anonymized.

### Analysis sets

To analyze the survey results, three analysis sets were formed. The first analysis set, titled ‘Overall Population of Practitioners,’ included all physicians who stated they treated patients in the indication area and prescribed at least one type of physiotherapy. Respondents who do not prescribe physiotherapy for either a meniscus lesion or ACL rupture were excluded from this analysis set, as it was assumed that these physicians do not generally prescribe physiotherapy. The second analysis set, titled ‘Prescribers for ACL Ruptures’, contains all physicians who stated they prescribe at least one type of physiotherapy for an ACL rupture. The third analysis set, titled ‘Prescribers for Meniscus Lesions’, includes all physicians who stated they prescribe at least one type of physiotherapy for a meniscus lesion. Respondents who did not provide information on the prescription of physiotherapy or stated that they do not treat either meniscus lesions or ACL ruptures were excluded from all analysis sets.

### Data analysis strategy

All analyses were descriptive in nature, as the objective of the study was to characterize current prescription practice rather than to test hypotheses or examine causal relationships. Continuous variables were summarized as mean (SD) and categorical variables as n (%). For each physiotherapy modality, the mean number of sessions was reported for the periods 0–6 months, 7–12 months, and cumulatively over 0–12 months, as well as the mean number of sessions considered desirable and the proportion of physicians prescribing the modality. Missing values were treated as missing and were not imputed (available-case analysis). No outlier removal was performed, as the full observed range was assumed to reflect real variation in clinical prescription practice. Analyses were performed in R (version 4.4.1).

## Results

### Sample description

The data collection of the online survey took place between May 28, 2024, and June 30, 2024, and was made available to physicians who are members of the professional associations of Arbeitsgemeinschaft Arthroskopie (AGA), Berufsverbands für Arthroskopie e.V. (BVASK) and Berufsverbands für Orthopädie und Unfallchirurgie e.V. (BVOU). A total of 800 individuals accessed the survey link, of whom 156 completed the questionnaire, resulting in a response rate of 19.5%. One of the respondents stated that they do not prescribe physiotherapy for either meniscus lesions or ACL ruptures; another stated that they do not treat patients with meniscus lesions or ACL ruptures. Three others did not provide information on the prescription of physiotherapy. The overall population of prescribers thus comprises *n* = 151 physicians. One additional participant stated that they do not prescribe physiotherapy for ACL ruptures, and *n* = 8 others stated that they do not prescribe physiotherapy for meniscus lesions. Thus, *n* = 150 participants are prescribers of physiotherapy for ACL ruptures and *n* = 143 are prescribers for meniscus lesions.

**Fig. 1:**
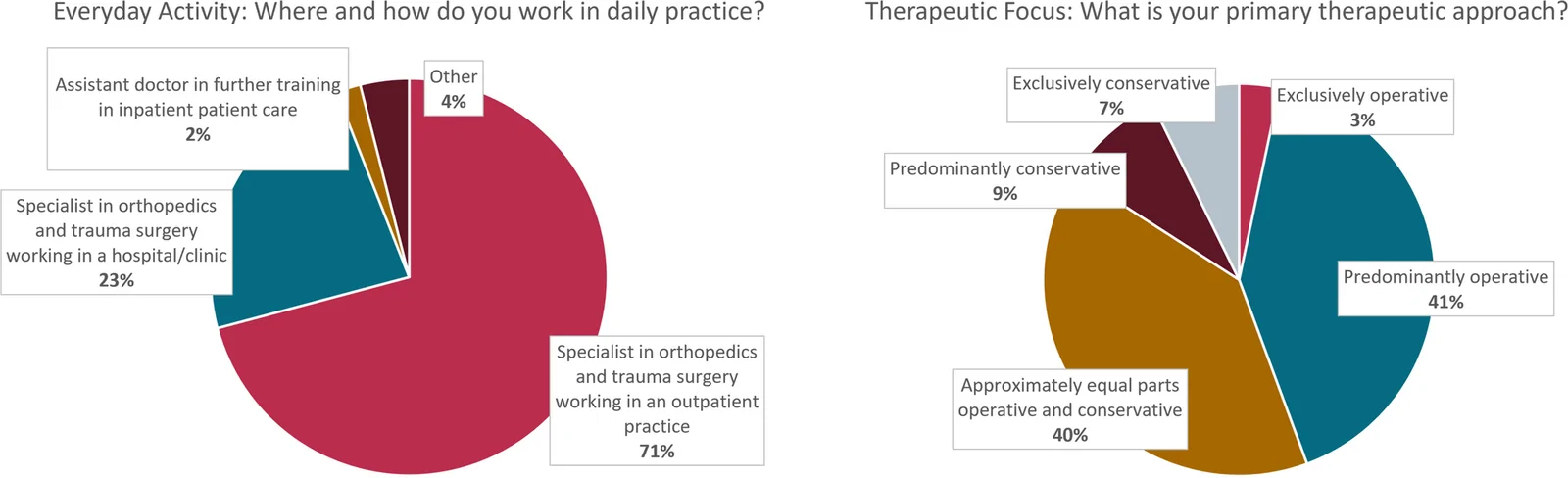
Professional fields and therapeutic focus of respondents

As shown in Fig. 1, of the *n* = 151 survey participants in the total population of prescribers, 107 (70.9%) are specialists in orthopaedics and trauma surgery working in an outpatient practice, 35 (23.2%) are specialists in orthopaedics and trauma surgery in a clinic, 3 (2%) are residents in inpatient care, and 6 (4%) have other professional activities. The therapy focuses of the participants are predominantly operative for 62 (41.1%) participants, equally in the operative and conservative areas for 60 (39.7%) participants, predominantly conservative for 13 (8.6%) participants, exclusively conservative for 11 (7.3%) participants, and exclusively operative for 5 (3.3%) participants.

### General care situation for an ACL rupture and a meniscus lesion

The survey examines the cooperation between clinics or practices and physiotherapists regarding the aftercare of patients following an ACL rupture or a meniscus lesion. Approximately half of the overall population of prescribers reported that patients receive a standardized aftercare plan from the clinics or practices, which the continuing physiotherapists follow, but without formal cooperation with them (49.3% of prescribers for ACL ruptures and 49% for meniscus lesions). Furthermore, 42% of prescribers reported that their facility has an aftercare concept for ACL rupture that is implemented in close cooperation with physiotherapists, while this was reported by 36.4% for meniscus lesions. A small proportion of prescribers leave the aftercare entirely to the treating physiotherapists (6% for ACL ruptures and 11.2% for meniscus lesions). 1.3% of prescribers stated that they could not assess the cooperation with physiotherapists for ACL rupture as part of aftercare. For meniscus lesions, this was the case for 1.4%.

The surveyed prescribers also provided information about their patients’ waiting times for a physiotherapy appointment. More than half reported waiting times of over two weeks, with 30.5% reporting a waiting time of 2 to 3 weeks and 27.2% reporting a waiting time of more than 3 weeks. About a quarter of the respondents (25.2%) reported waiting times of 1 to 2 weeks. Only a minority of 15.9% stated that patients receive an appointment within one week. Furthermore, most prescribers stated that they never (61.6%) or rarely (15.9%) schedule surgery dates depending on the availability of outpatient postoperative physiotherapy.

### Physiotherapeutic care for patients with ACL rupture in Germany

The survey initially assesses the current state of physiotherapeutic care for patients with ACL ruptures, as well as the surveyed physicians’ assessments of ideal or desired care. On average, physicians prescribing physiotherapy for ACL ruptures manage approximately 96 ACL rupture cases per year (SD = 108, median = 50) with caseloads ranging from 3 to 650 cases.

The physicians provided information on the number of prescribed sessions for each type of physiotherapy (therapeutic exercises, device-based physiotherapy, manual lymphatic drainage, manual therapy, and other therapies).

**Fig. 2:**
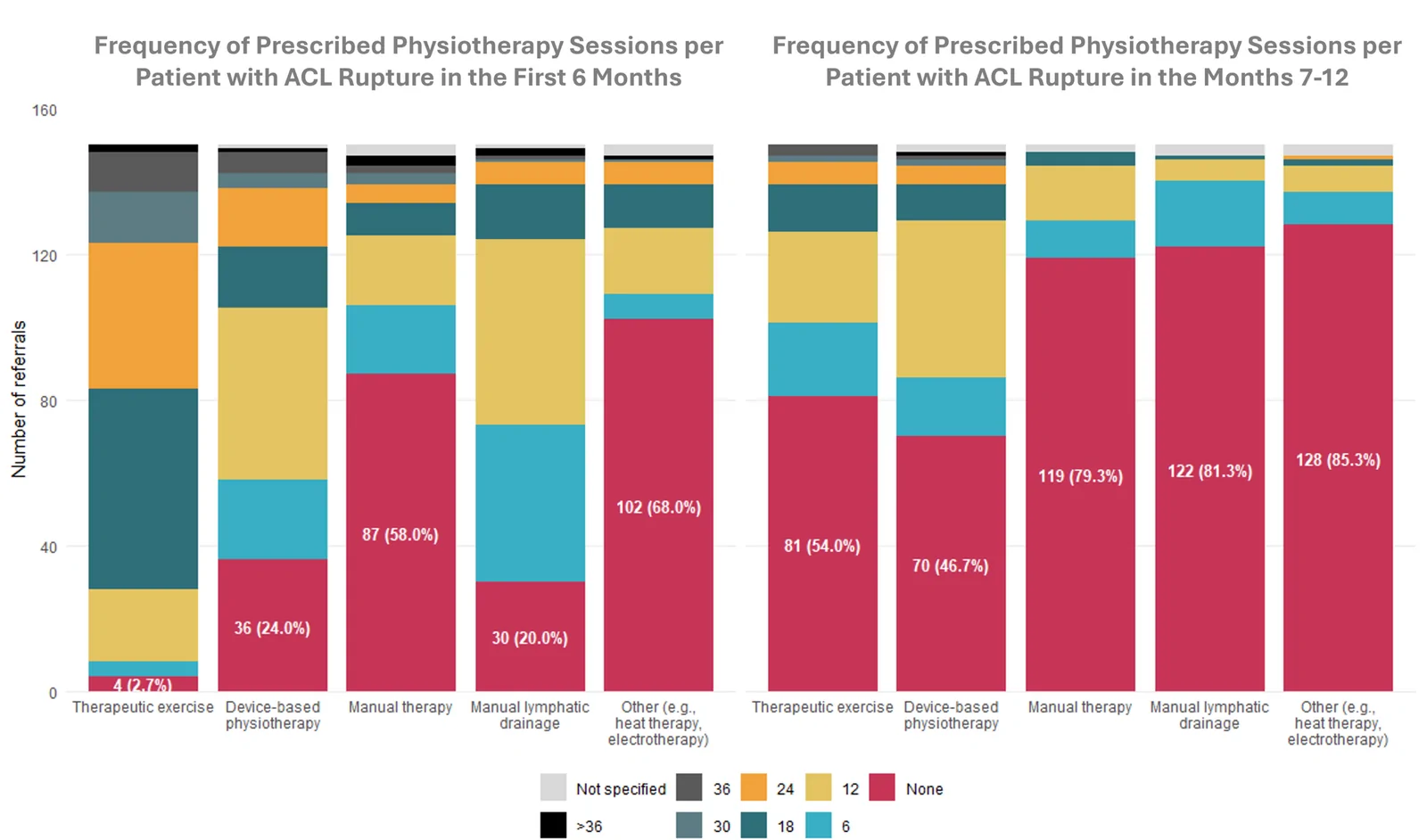
Physiotherapy prescriptions after ACL rupture: 0–6 months vs. 7–12 months

As depicted in Fig. 2, therapeutic exercise is the predominant physiotherapeutic modality prescribed in the first six months after ACL rupture, with 97.3% of respondents indicating its utilization. This is followed by manual lymphatic drainage (80.0%) and device-based physiotherapy (76.0%). Manual therapy is employed by 42.0%, and other therapeutic interventions by 32.0% of the respondents during this period. Subsequently, within the 7- to 12-month post-injury interval, a shift in prescription patterns can be observed. Device-based physiotherapy emerge as the most frequently prescribed modality (53.3%), followed by therapeutic exercises (46.0%). Manual therapy is prescribed by 21.7% of physicians, manual lymphatic drainage by 18.7%, and adjunctive therapies, such as thermotherapy and electrotherapy, by 14.7% of respondents.

**Fig. 3:**
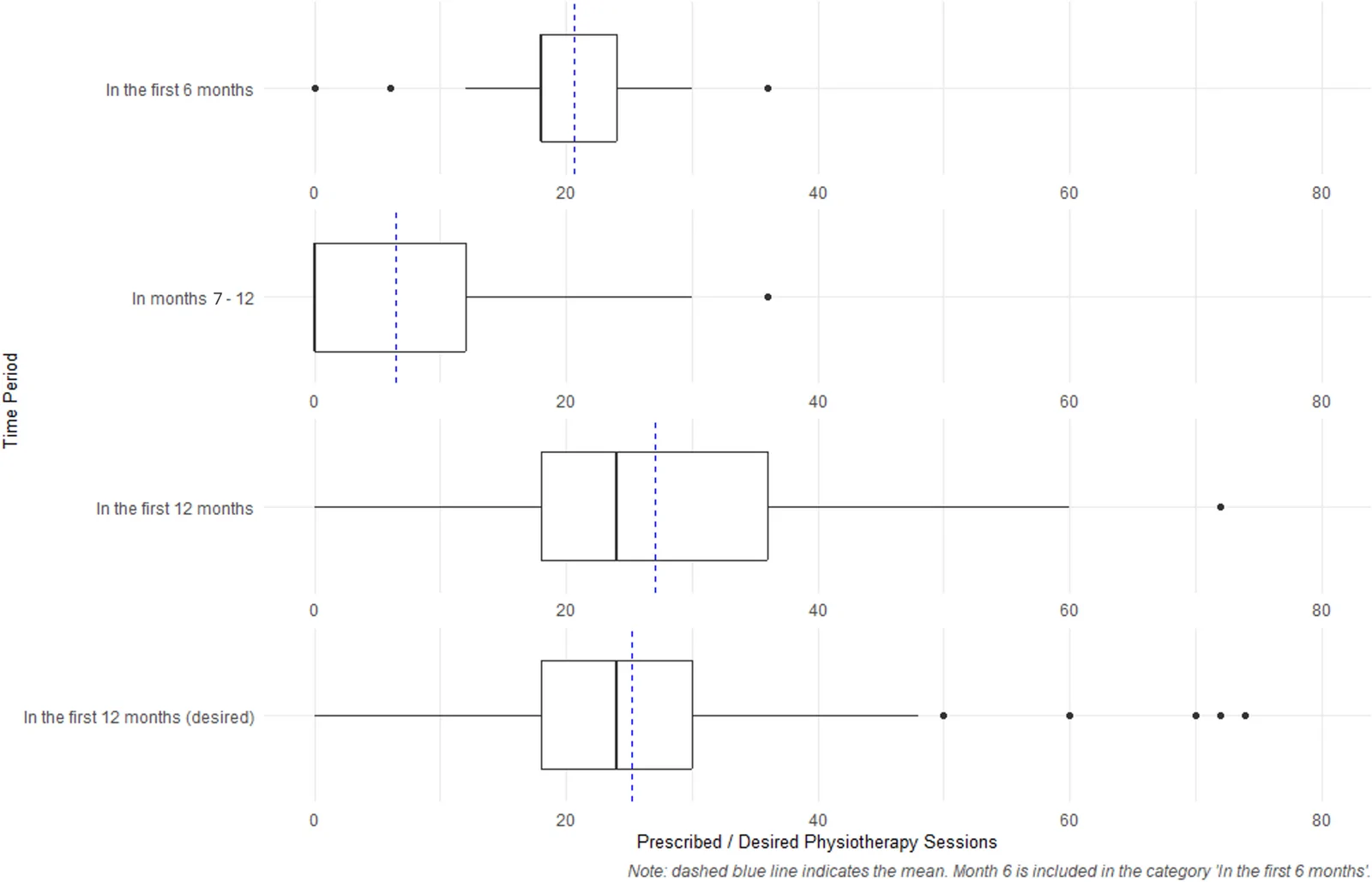
Prescribed and desired sessions of therapeutic exercises after ACL rupture

On average, within the initial six months following an ACL rupture, 20.7 sessions of therapeutic exercise (SD = 7.9; median = 18), the most frequently used physiotherapeutic modality, are prescribed. The reported values range from 0 to 36 sessions. During the subsequent 6-month period, the mean number of sessions decreased to 6.4 (SD = 8.8; median = 0), with a range of 0 to 36 sessions. Cumulatively, an average of 27.1 sessions of therapeutic exercise are prescribed within the first year (SD = 13.6; median = 24), with values ranging from 0 to 72 sessions. The desired number of sessions, as indicated by the surveyed physicians, shows a similar distribution. Specifically, they prefer a mean of 25.2 sessions (SD = 12.8; median = 24), with a range of 0 to 74 sessions. These distributions are visually presented in Fig. 3 using box plots. The amount of the additional prescribed modalities and the difference compared to the desired amounts are listed in Table 1.

**Table 1:** Prescription practice of physiotherapeutic modalities and desired amount after ACL rupture

Modalities	Prescribed (0–6 months)	Prescribed (7–12 months)	Prescribed Total (0–12 months)	Desired Total (0–12 months)	∆_Prescribed−Desired_
TE	20.7 ± 7.9	6.4 ± 8.8	27.1 ± 13.6	25.2 ± 12.8	1.9
DBP	11.8 ± 9.8	7.1 ± 8.2	18.9 ± 15.2	18.4 ± 14.3	0.5
MT	6.1 ± 9.4	2.1 ± 4.6	8.2 ± 12.3	7.9 ± 11.0	0.3
MLD	9.5 ± 7.5	1.3 ± 3.3	10.9 ± 9.3	9.9 ± 7.7	1.0
Other	4.7 ± 7.9	1.3 ± 4.0	6.0 ± 10.6	5.6 ± 10.1	0.4

TE = Therapeutic exercise; DBP = Device-based physiotherapy; MT = Manual therapy; MLD = Manual lymphatic drainage

In addition to therapeutic exercise, an average of 11.8 sessions of device-based physiotherapy is prescribed in the first six months after ACL rupture (SD = 9.8; median = 12; min. = 0; max. = 36). During the subsequent six months, the mean prescription decreases to 7.1 sessions (SD = 8.2; median = 6; min. = 0; max. = 36), resulting in a cumulative prescription of 18.9 sessions within the first year (SD = 15.17; median = 18; min. = 0; max. = 72). The surveyed physicians indicated a desired average of 18.4 sessions of device-based physiotherapy (SD = 14.3; median = 12; min. = 0; max. = 80).

Regarding manual therapy, an average of 6.1 sessions is prescribed within the initial six months (SD = 9.4; median = 0; min. = 0; max. = 36). During the 7–12 months interval, the mean decreases to 2.1 sessions (SD = 4.6; median = 0; min. = 0; max. = 18), resulting in a cumulative prescription of 8.2 sessions within the first year (SD = 12.3; median = 0; min. = 0; max. = 54). The physicians’ desired mean was 7.9 sessions (SD = 11; median = 5.5; min. = 0; max. = 52).

For manual lymphatic drainage, an average of 9.5 sessions is prescribed within the initial six months post-ACL rupture (SD = 7.5; median = 12; min. = 0; max. = 36). During the 6- to 12-month interval, this value decreases to 1.3 sessions (SD = 3.3; median = 0; min. = 0; max. = 18), resulting in a cumulative prescription of 10.9 sessions within the first year (SD = 9.3; median = 12; min. = 0; max. = 54). The physician’s desired average is 9.9 sessions (SD = 7.7; median = 10; min. = 0; max. = 36).

For other physiotherapeutic modalities, an average of 4.7 sessions is prescribed within the initial six months (SD = 7.9; median = 0; min. = 0; max. = 36), and 1.3 sessions during the subsequent six months (SD = 4; median = 0; min. = 0; max. = 24). Cumulatively, an average of 6 sessions is prescribed within the first year (SD = 10.6; median = 0; min. = 0; max. = 48). The surveyed physicians indicate a desired mean of 5.6 sessions for the treatment of ACL ruptures (SD = 10.1; median = 0; min. = 0; max. = 50).

Regarding the treatment of ACL ruptures, the frequency of preoperative physiotherapy prescription in cases with extended intervals between injury and surgery was also assessed. The majority of physicians managing ACL ruptures reported consistently (34%) or frequently (30.7%) prescribing such preoperative physiotherapy. The remaining respondents indicated infrequent (14.7%), no (5.3%), or occasional (15.3%) prescription. Furthermore, the frequency of referral to full-day outpatient or inpatient rehabilitation programs for ACL rupture patients was investigated. However, the majority of physicians treating ACL ruptures reported infrequent (37.3%) or no (20%) referrals for such rehabilitative measures.

In the ACL cohort, referral patterns differed according to the physicians’ treatment profile (see Table 2). Physicians with an operative focus reported the highest mean number of referrals for therapeutic exercise (TE: 35.6 ± 23.8) and device-based physiotherapy (DBP: 26.6 ± 18.0). In contrast, predominantly conservative physicians reported substantially lower values (TE: 21.7 ± 10.7 and DBP: 13.7 ± 10.9), as did conservative physicians (TE: 15.5 ± 7.9 and DBP: 15.5 ± 11.9). A similar trend was observed for manual therapy and other modalities, with referrals generally being highest among operative physicians and lowest among conservative physicians (e.g. manual therapy: 24.0 ± 18.2 vs. 6.4 ± 8.0; other modalities: 9.6 ± 19.2 vs. 1.0 ± 3.4). Referrals for manual lymphatic drainage (MLD) showed smaller differences between treatment profiles, ranging from 7.8 ± 5.2 to 15.6 ± 8.1.

**Table 2:** Treatment profile and referral modalities of participating physicians in the ACL cohort

Modalities	Operative	Predominantly operative	Both	Predominantly conservative	Conservative
TE	35.6 ± 23.8	25.8 ± 13.8	24.7 ± 10.9	21.7 ± 10.7	15.5 ± 7.9
DBE	26.6 ± 18.0	21.7 ± 16.0	13.9 ± 11.6	13.7 ± 10.9	15.5 ± 11.9
MT	24 ± 18.2	9.2 ± 10.4	5.4 ± 10.2	4.6 ± 4.8	6.4 ± 8.0
MLD	15.6 ± 8.1	9.7 ± 8.2	9.6 ± 7.1	8.9 ± 7.9	7.8 ± 5.2
Other	9.6 ± 19.2	6.7 ± 11.1	4.2 ± 7.6	5.2 ± 7.9	1.0 ± 3.4

TE = Therapeutic exercise; DBP = Device-based physiotherapy; MT = Manual therapy; MLD = Manual lymphatic drainage

### Physiotherapeutic management of meniscal lesions in Germany

Within the scope of this investigation, the current status of physiotherapeutic management of meniscal lesions in Germany. The physicians surveyed who prescribe physiotherapy for meniscal lesions reported managing an average of approximately 280 cases annually (SD = 323.7; median = 200), with caseloads ranging from 10 to 2000 cases.

**Fig. 4:**
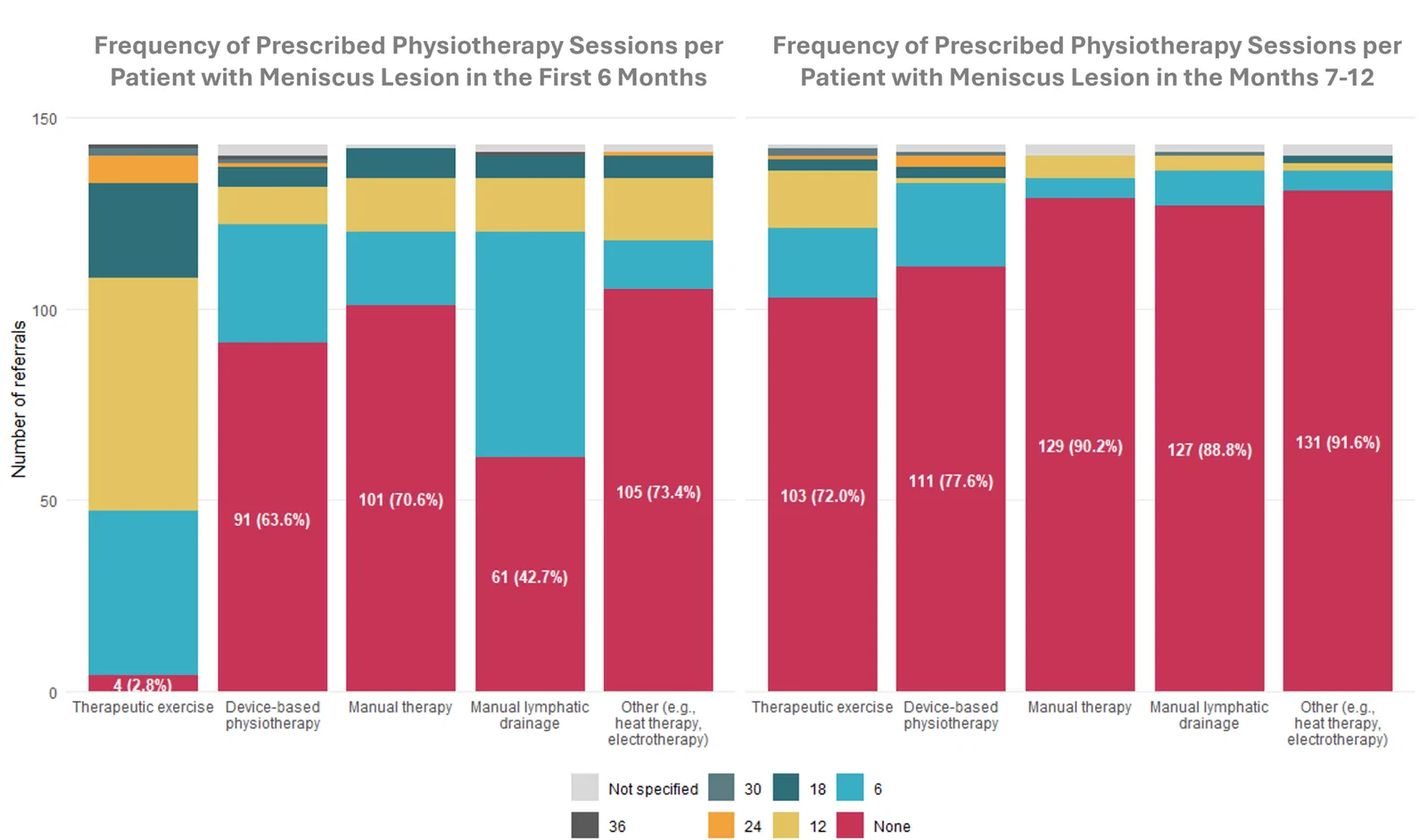
Physiotherapy prescriptions after meniscus lesion: 0–6 months vs. 7–12 months

The surveyed physicians were queried regarding the number of prescribed sessions for each physiotherapeutic modality (therapeutic exercises, device-based physiotherapy, manual lymphatic drainage, manual therapy, and other therapies). As depicted in Fig. 4, therapeutic exercise is the most frequently prescribed physiotherapeutic modality within the initial six months following a meniscal lesion; at least 6 sessions are prescribed by 97.2% of the surveyed physicians. Manual lymphatic drainage is the second most frequently prescribed modality (57.3%), followed by device-based physiotherapy (36.4%). Manual therapy is prescribed by 28.7%, and other therapeutic interventions by 25.2% of the respondents. During the subsequent 7–12 month period, therapeutic exercise remains the most frequently prescribed modality, although at a reduced rate of 28.0% of respondents. Device-based physiotherapy is prescribed by 22.4% of respondents in this phase, followed by manual lymphatic drainage (11.2%), manual therapy (9.8%), and other therapeutic interventions (8.4%).

**Fig. 5:**
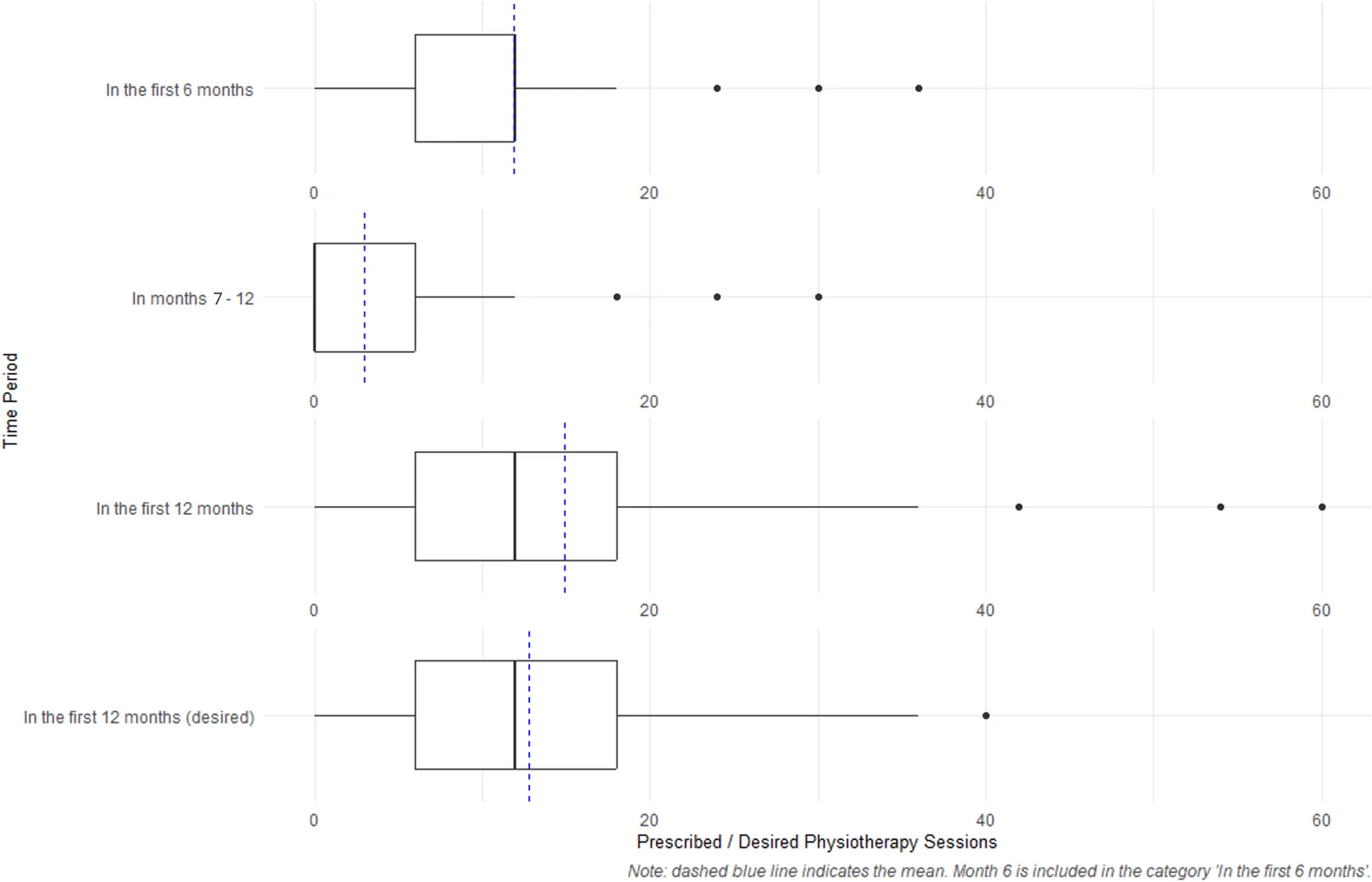
Prescribed and desired sessions for therapeutic exercises after meniscus lesion

On average, within the initial six months following a meniscal lesion, 11.9 sessions of therapeutic exercises, the most frequently prescribed physiotherapeutic modality, are performed (SD = 6.1; median = 12), with values ranging from 0 to 36 sessions. During the subsequent six months, the mean number of sessions decreases to 3 sessions (SD = 5.9; median = 0), with a range of 0 to 30 sessions. Cumulatively, an average of 14.9 sessions of therapeutic exercise is prescribed during the first year (SD = 10; median = 12), with values ranging from 0 to 60 sessions. The desired number of sessions, as indicated by the surveyed physicians, shows similar distributions: the mean desired value is 12.8 sessions of therapeutic exercise (SD = 7.2; median = 12), with responses ranging from 0 to 40 sessions. These distributions are visually presented in Fig. 5 using box plots. The amount of the additional prescribed modalities and the difference compared to the desired amounts are listed in Table 3.

**Table 3:** Prescription practice of physiotherapeutic modalities and desired amount after meniscus lesion

Modalities	Prescribed (0–6 months)	Prescribed (7–12 months)	Prescribed Total (0–12 months)	Desired Total (0–12 months)	∆_Prescribed−Desired_
TE	11.9 ± 6.1	3.0 ± 5.9	14.9 ± 10.0	12.8 ± 7.2	2.1
DBP	3.5 ± 6.2	2.1 ± 5.3	5.6 ± 10.3	6.0 ± 7.9	-0.4
MT	3.0 ± 5.3	0.7 ± 2.6	3.7 ± 7.0	3.6 ± 5.7	0.1
MLD	4.7 ± 5.5	0.9 ± 3.5	5.6 ± 8.1	4.7 ± 5.3	0.9
Other	2.9 ± 5.4	0.6 ± 2.8	3.4 ± 7.2	2.7 ± 5.5	0.7

TE = Therapeutic exercise; DBP = Device-based physiotherapy; MT = Manual therapy; MLD = Manual lymphatic drainage

In addition to therapeutic exercise, an average of 3.5 sessions of device-based physiotherapy are prescribed within the initial six months following a meniscal lesion (SD = 6.2; median = 0; min. = 0; max. = 36). During the subsequent six months, the mean number of sessions decreases to 2.1 sessions (SD = 5.3; median = 0; min. = 0; max. = 30), resulting in a cumulative prescription of 5.6 sessions within the first year (SD = 10.3; median = 0; min. = 0; max. = 60). The surveyed physicians indicated a desired mean of 6 sessions of device-based physiotherapy (SD = 7.9; median = 6; min. = 0; max. = 40).

Regarding manual therapy, an average of 3 sessions are prescribed within the initial six months following a meniscal lesion (SD = 5.3; median = 0; min. = 0; max. = 18). During the 7–12 month period, the mean decreases to 0.7 sessions (SD = 2.6; median = 0; min. = 0; max. = 12), resulting in a cumulative prescription of 3.7 sessions within the first year (SD = 7; median = 0; min. = 0; max. = 30). The mean desired value of the physicians is 3.6 sessions (SD = 5.7; median = 0; min. = 0; max. = 20).

For manual lymphatic drainage, an average of 4.7 sessions are prescribed within the initial six months following a meniscal lesion (SD = 5.5; median = 6; min. = 0; max. = 36). During the 7–12 month period, the prescription rate decreases to 0.9 sessions (SD = 3.5; median = 0; min. = 0; max. = 30), resulting in a cumulative prescription of 5.6 sessions within the first year (SD = 8.1; median = 6; min. = 0; max. = 66). The desired average number of sessions is 4.7 sessions (SD = 5.3; median = 6; min. = 0; max. = 40).

For other physiotherapeutic modalities, an average of 2.9 sessions were prescribed in the first six months after a meniscal lesion (SD = 5.4; median = 0; min. = 0; max. = 24). During the following six months, the mean prescription decreases to 0.6 sessions (SD = 2.8; median = 0; min. = 0; max. = 18), resulting in a cumulative prescription of 3.4 sessions within the first year (SD = 7.2; median = 0; min. = 0; max. = 42). For the entire post-meniscal lesion period, the respondents indicated a desired mean of 2.7 sessions (SD = 5.5; median = 0; min. = 0; max. = 30).

In the meniscus cohort, referral modalities again demonstrated a clear preference for conventional physical therapy across all physician treatment profiles (see Table 4). On average, physicians reported 12.2 ± 7.5 referrals for therapeutic exercise (TE), with slightly higher values among purely operative physicians (14.4 ± 4.8) and those who were predominantly operative (12.7 ± 9.2), compared with those who were predominantly conservative (10.9 ± 4.0) or conservative (10.6 ± 3.0). Referrals for device-based physiotherapy (DBP) were less frequent overall (5.8 ± 7.8), but showed a distinct pattern: markedly lower use was observed among purely operative physicians (1.2 ± 2.4), compared with all other profiles (3.1–7.6). Manual therapy and manual lymphatic drainage were prescribed at similarly low to moderate levels across treatment profiles (MT: 3.4 ± 5.6; MLD: 4.5 ± 5.3).

**Table 4:** Treatment profile and referral modalities of participating physicians in the meniscus cohort

Modalitites	Operative	Predominantly operative	Both	Predominantly conservative	Conservative
TE	14.4 ± 4.8	12.7 ± 9.2	11.4 ± 7.0	10.9 ± 4.0	10.6 ± 3
DBP	1.2 ± 2.4	7.6 ± 9	4.5 ± 6.7	4.2 ± 4.3	3.1 ± 5.7
MT	6.8 ± 6.8	4.3 ± 5.7	2.1 ± 5.2	2.8 ± 3.8	2.5 ± 4.6
MLD	4.8 ± 4.5	4.9 ± 6.0	4.1 ± 5.0	4.1 ± 3.6	2.6 ± 2.9
Other	3.6 ± 7.2	3.0 ± 5.8	2.1 ± 4.8	3.2 ± 5.1	0 ± 0

TE = Therapeutic exercise; DBP = Device-based physiotherapy; MT = Manual therapy; MLD = Manual lymphatic drainage

## Discussion

### Summary of the research question and main results

This study was designed as a descriptive survey to characterize current prescription practices after ACL rupture and meniscal lesions from the perspective of treating physicians in Germany. It does not aim to establish causal relationships or evaluate treatment effectiveness. Instead, the findings highlight how physicians translate clinical guidelines and structural care constraints into actual prescriptions. As such, the results provide insight into clinical decision-making processes and potential gaps between guideline recommendations and real-world practice.

Specialists from both outpatient practices and hospital settings participated in the survey, representing a broad spectrum of orthopedic and trauma surgery prescribing patterns. As expected, similar trends regarding the prescribed therapeutic modalities emerged for both indications, aligning with the therapeutic objectives described in the literature [9, 18]. At the initiation of rehabilitation, the focus should be on reducing oedema and improving mobility, while muscle strengthening should be prioritized in subsequent phases [9, 18]. Thus, therapeutic exercises and manual lymphatic drainage are the most frequently prescribed therapeutic modalities for both injuries within the initial six months. In contrast, during the subsequent 7–12 month period, therapeutic exercises and device-based physiotherapy are the most frequently prescribed treatments. Manual therapy is prescribed less frequently overall for both indications and, when prescribed, it is typically administered during the early rehabilitation phase. Other therapeutic modalities play a subordinate role in the injuries examined and are prescribed by a minority of the participating physicians. Unlike the survey results reported by Nolte and Fleßa for general physiotherapy prescription in Germany [13], the prescribing practices for the investigated indications do not focus mainly on physiotherapy and manual therapy, but encompass a broader range of therapeutic modalities, which may allow the specific key symptoms to be addressed more effectively.

### Interpretation of the prescription practice

The number of physiotherapy sessions prescribed decreases over time. For example, therapeutic exercises, the most frequently employed therapy after ACL rupture, are prescribed at an average of 20.7 sessions in the initial six months, but only 6.4 sessions in the subsequent six months. More than half of the physicians do not prescribe any therapeutic exercise at all in the 7–12 month postoperative period. The increasing variability in prescription volume is also reflected in the widening discrepancies between the mean and median during the second half of the year. A possible explanation is that orientation toward indication-specific guidelines is more widely followed by physicians during the early stage of rehabilitation. In total, 27.1 sessions are prescribed within the first year, exceeding the 18 sessions recommended in the German catalogue of therapies. The actual number of sessions prescribed is very close to the number of physicians felt would be appropriate (25.2 sessions), highlighting a significant need. A similar reduction in prescribed sessions is observed for meniscal lesions. While the number of therapeutic exercise sessions prescribed in the first year (14.9 sessions) corresponds with the desired value (12.8 sessions), this prescription volume is within the range recommended in the German catalogue of therapies. The differences in prescribing practice for therapeutic exercises for the two indications in this study show that the need for physiotherapy support varies greatly depending on the indication and should be determined accordingly. Based on international guidelines regarding the rehabilitation duration for both indications and the time until return to sports activity, it was expected that the extent of physiotherapy prescriptions for cruciate ligament ruptures would be higher than for meniscus lesions [10, 16]. This is consistent with the findings of a study from the United States that surveyed physiotherapists regarding the optimal amount of physiotherapy required for full recovery from various orthopedic conditions [19]. Similar to the present survey, the range of responses was very broad, and the estimated need after ACL reconstruction (37.3 sessions) appears to be higher than after meniscus repair (23.7 sessions) [19].

Demand for device-based physiotherapy is particularly high in the treatment of ACL ruptures. The distribution of this modality over the first year is more homogeneous, with an average of 18.9 sessions prescribed, which aligns with the number deemed appropriate by the physicians. The variability in the provision of care, reflecting individual prescriber preferences, is exemplified by the use of manual therapy in the physiotherapy of patients following ACL rupture. While 60% of respondents do not prescribe manual therapy at all, some prescribe an average of 54 sessions within the first year. A wide range in the number of prescribed sessions is also evident in meniscal lesions. However, it should be noted that the survey did not differentiate between meniscal repair and meniscectomy. Due to the typically longer period of partial weight-bearing and movement restriction associated with meniscal repair, a longer rehabilitation period is generally expected.

For both indicated conditions, the rehabilitation process is extensive and structured in phases [9, 18, 20], which should be addressed through a combination of various therapeutic modalities. Considering that over 40% of physicians prescribe more than 24 sessions of therapeutic exercises alone in the first 6 months following an ACL rupture, the combination with additional therapeutic modalities significantly exceeds the recommended 18 sessions. There is hardly any adequate reference of physiotherapy utilization in the literature for an international comparison. No studies could be found that differentiate between the various physiotherapy modalities. One study from Poland reports a distinction between groups with fewer or more than 60 supervised physiotherapy sessions within the first six months after ACL rupture indicating an extensive rehabilitation amount [21]. Le Sant et al. report a similar level based on real-world data from the French healthcare system, with a mean of 15.2 pre-operative and 46.8 post-operative sessions [22]. In contrast, Fausett et al. report that in New Zealand, 81% of the studied cohort received fewer than 15 physiotherapy sessions after cruciate ligament rupture [23]. The overall combination of different physiotherapy modalities in Germany is consistent with the levels reported in other European countries, even though the German catalogue of therapies specifies a lower number of sessions.

Our findings on ACL and meniscus injuries show that, although traditional physical therapy is common in both cohorts, the frequency of referrals and the use of equipment-based procedures depend significantly on the treatment profile of the physicians (operative, conservative, or both). One possible explanation is that surgeons are more likely to treat patients with a greater symptom burden or more complex lesions, who they perceive to have a greater need for intensive, structured rehabilitation. Another possible explanation is that, in the context of surgery, physicians may aim to protect the surgical outcome by prescribing more supervised therapy. However, these assumptions remain speculative as we did not systematically assess the clinical rationale behind individual referrals or the case mix of the different physician groups. This represents an important limitation when interpreting the observed differences in referral patterns.

### Interpretation of the availability of physiotherapy

Concurrently, over half of the respondents indicated average waiting times exceeding two or three weeks for scheduled physiotherapy appointments. It is plausible that regional disparities between urban and rural locales may contribute to further extended waiting periods. A report issued by the Federal Association for Physiotherapy suggests that patients requiring home-based therapeutic interventions frequently experience more protracted waiting times [24]. Both aspects require further investigation in future research endeavors. In all instances, the prompt initiation of therapy serves as a critical determinant of positive therapeutic outcomes [9, 16, 25]. Furthermore, waiting times can delay rehabilitation and thus the return to sport or work. In specific cases, such as after arthrolysis, immediate physiotherapy is crucial [26].

Potential for optimization exists, among other things, in improved surgical planning management. Over 75% of prescribers indicate that they never or rarely schedule surgery dates according to the availability of outpatient postoperative physiotherapy. In view of the frequently non-urgent and elective nature of these surgeries, interdisciplinary coordination could minimize waiting times and thereby improve postoperative outcomes. Although it has been shown that preoperative strength deficits or range of motion limitations can lead to inferior postoperative outcomes after ACL rupture [16], only 64.7% of respondents prescribe preoperative physiotherapy.

### Strengths and limitations

When considering the results, it is important to note that the data was collected through an online survey, and thus no clarification questions regarding the items could be asked. Furthermore, of the 800 people contacted, only 156 completed the questionnaire up to the last page. However, participation in the questionnaire was voluntary, and no advantage accrued to the participants. Therefore, it can be assumed that there was an intrinsic motivation to contribute to this relevant topic, potentially leading to a self-selection bias. As participation was anonymous, no comparison between respondents and non-respondents was possible. Therefore, the representativeness of the sample to the broader population of physiotherapy prescribers cannot be fully ensured. For similar future surveys, reminders should be implemented to increase the response rate.

Due to the recruitment method, almost exclusively orthopedic and trauma surgeons were surveyed, while other prescribers such as general practitioners were not reached. The distribution of the survery was carried out through the professional associations, which may have introduced a selection bias and limits the generalizability of the results. As approximately 40% of physiotherapy treatments are prescribed by general practitioners and primary care physicians [27], these should also be surveyed in the future. A further limitation is the high variability in the number of cases by individual physicians. Since prescribing practices and the number of prescriptions are closely linked to a country’s healthcare system, these data are representative only for Germany and should be compared with other countries in future studies. Since the responses are self-reported by the physicians, a bias due to socially desirable answers cannot be ruled out, particularly regarding the relationship between the desired number of physiotherapy sessions and the number actually prescribed.

Additionally, the study did not differentiate between isolated and combined injuries, even though the latter could necessitate a significantly larger scope of rehabilitation due to their higher severity. Furthermore, no distinction was made between the different surgical procedures for meniscal injuries. Since the duration and intensity of rehabilitation differ significantly between these procedures, this should be investigated more closely in future studies.

A further limitation of this study is that the survey did not explicitly address the underlying drivers of current prescription practices. Future research should therefore investigate whether indication-specific guidelines, individual patient needs, or physician routine are the decisive factors determining the number of prescribed therapy units.

Nevertheless, the survey provides a comprehensive insight into current prescription practices of physiotherapy for ACL ruptures and meniscal lesions in Germany and identifies potential areas for optimization. In the future, the prescription practices of other indications should be analyzed, and a more detailed stratification according to the surgical pathways should be included.

### Implications for practice, research and policy

Overall, prescribing practices are highly heterogeneous and reveal that the German catalogue of therapies and the clinical guidelines do not provide a clear framework for rehabilitation care. In the future, these should be standardized to ensure that patients receive a minimum level of rehabilitation and to prevent secondary complications. The findings of this study indicate that the specifications in the German catalogue of therapies lack practical relevance, as both the perceived adequate amount and the actual number of prescribed sessions exceed these regulations. To ensure that the catalogue can serve as a meaningful guide in the future, its recommendations should be indication-specific, as significant differences were observed even between ACL and meniscus rehabilitation. Furthermore, aligning rehabilitation guidelines with the catalogue of therapies would be desirable, allowing for specific recommendations for individual physiotherapeutic modalities. If expanding the current regulatory framework is not feasible, one potential approach involves complementary digital health applications, which could help absorb the excess care burden and contribute to a more consistent quality of rehabilitation across individuals.

For research, the study identifies a need for further work using routine healthcare data (e.g., health insurance claims, physiotherapy documentation systems) to validate the reported prescription patterns and to assess their association with functional outcomes. As the study relies on self-reported data, it cannot verify whether the prescriptions correspond to the exact number of physiotherapy sessions delivered or completed. Future research should therefore combine survey data with routine care data to improve comparability and to examine patient outcomes.

## Conclusion

The survey reveals both similarities and differences in the physiotherapeutic prescription practices for patients after ACL rupture or meniscal lesions. The actual prescribed volume of the most frequently prescribed modality, therapeutic exercises, significantly exceeds the 18 sessions indicated in the German catalogue of therapies for rehabilitation of patients after ACL rupture. This, in combination with additional therapeutic modalities, underlines a substantial need for physiotherapy follow-up. This is contrasted by frequent waiting times for therapy appointments and, consequently, a disrupted therapeutic course. One potential approach to addressing this care gap could involve digital health applications, which might support independent training of the affected individuals. In addition to improved interdisciplinary planning of post-operative aftercare, exploring such digital opportunities represents one way to potentially meet these demands.

## Data Availability

Data are available upon reasonable request from the corresponding author.

## References

[1] Rommel AP (2017) F., Inanspruchnahme physiotherapeutischer Leistungen in Deutschland. Journal of Health Monitoring

[2] Schneider O et al (2016) [Incidence of knee injuries: Numbers for outpatient and inpatient care in Germany]. Orthopade 45(12):1015–1026 10.1007/s00132-016-3301-6 27518117

[3] Ardern CL et al (2014) Fifty-five per cent return to competitive sport following anterior cruciate ligament reconstruction surgery: an updated systematic review and meta-analysis including aspects of physical functioning and contextual factors. Br J Sports Med 48(21):1543–1552 10.1136/bjsports-2013-093398 25157180

[4] Paterno MV et al (2017) Clinical Factors That Predict a Second ACL Injury After ACL Reconstruction and Return to Sport: Preliminary Development of a Clinical Decision Algorithm. Orthop J Sports Med 5(12):2325967117745279 10.1177/2325967117745279 PMC5753959 29318172

[5] Schilaty ND et al (2017) Incidence of Second Anterior Cruciate Ligament Tears and Identification of Associated Risk Factors From 2001 to 2010 Using a Geographic Database. Orthop J Sports Med, 5(8) 10.1177/2325967117724196 PMC5564962 28840155

[6] Wiggins AJ et al (2016) Risk of Secondary Injury in Younger Athletes After Anterior Cruciate Ligament Reconstruction: A Systematic Review and Meta-analysis. Am J Sports Med 44(7):1861–1876 10.1177/0363546515621554 PMC5501245 26772611

[7] Poulsen E et al (2019) Knee osteoarthritis risk is increased 4–6 fold after knee injury - a systematic review and meta-analysis. Br J Sports Med 53(23):1454–1463 10.1136/bjsports-2018-100022 31072840

[8] DGOU, *Nachbehandlungsempfehlungen 2022 - Arbeitskreis Traumarehabilitation | Sektion Physikalische Therapie und Rehabilitation der DGOU*. (2022) Deutsche Gesellschaft für Orthopädie und Unfallchirurgie e.V. p. 156

[9] Filbay SR, Grindem H (2019) Evidence-based recommendations for the management of anterior cruciate ligament (ACL) rupture. Best Pract Res Clin Rheumatol 33(1):33–47 10.1016/j.berh.2019.01.018 PMC6723618 31431274

[10] Pujol N et al (2025) The Formal EU-US Meniscus Rehabilitation 2024 Consensus: An ESSKA-AOSSM-AASPT Initiative: Part I-Rehabilitation Management After Meniscus Surgery (Meniscectomy, Repair and Reconstruction). Orthop J Sports Med 13(5):23259671251343088 10.1177/23259671251343088 PMC12099113 40416991

[11] Prill R et al (2025) The formal EU-US Meniscus Rehabilitation 2024 Consensus: An ESSKA-AOSSM-AASPT initiative. Part II-Prevention, non-operative treatment and return to sport. Knee Surg Sports Traumatol Arthrosc 33(8):3014–3024 10.1002/ksa.12689 PMC12310080 40501314

[12] Bundesausschuss G (2024) Richtlinie des Gemeinsamen Bundesausschusses über die Verordnung von Heilmitteln in der vertragsärztlichen Versorgung (Heilmittel-Richtlinie/HeilM-RL). Bundesanzeiger

[13] Nolte R, Fleßa S (2016) Umsetzung der Heilmittel-Richtlinie in der Physiotherapie. HeilberufeScience 7(4):132–142

[14] Herbort PDM, Lobenhoffer PDP (2018) DGU-Leitlinie 012 – 005 Vordere Kreuzbandruptur. Leitlinienkommission der Deutschen Gesellschaft für Unfallchirurgie e.V, p 36

[15] Siebert CH et al (2018) [S2k-Guideline on Meniscal Disease: Non-operative and Surgical Management]. Z Orthop Unfall 156(3):324–329 10.1055/s-0044-101558 29529695

[16] van Melick N et al (2016) Evidence-based clinical practice update: practice guidelines for anterior cruciate ligament rehabilitation based on a systematic review and multidisciplinary consensus. Br J Sports Med 50(24):1506–1515 10.1136/bjsports-2015-095898 27539507

[17] Monson JK et al (2025) Current Rehabilitation Principles Following Meniscus Repairs. Curr Rev Musculoskelet Med 18(9):331–343 10.1007/s12178-025-09967-6 PMC12283527 40343689

[18] Keller H et al (2020) Empfehlungen zur postoperativen Rehabilitation nach Meniskusverletzungen. Orthopädische und Unfallchirurgische Praxis 9(2):116–120

[19] Pyrz K et al (2024) Does Insurance Provide Adequate Coverage for Physical Therapy Visits for Common Orthopedic Diagnoses? A Survey Study. South Med J 117(7):353–357 10.14423/SMJ.0000000000001707 38959960

[20] Zumstein F et al (2019) Kombinierte zeit- und kriterienbasierte Rehabilitation nach Rekonstruktion des vorderen Kreuzbandes. Sports Orthop Traumatol 35(2):130–141

[21] Czamara A et al (2021) The Muscle Strength of the Knee Joint after ACL reconstruction depends on the number and frequency of supervised physiotherapy visits. Int J Environ Res Public Health, 18(20) 10.3390/ijerph182010588 PMC8536018 34682334

[22] Le Sant G et al (2025) Effects of preoperative treatment on healthcare utilization and return to work for anterior cruciate ligament injuries: a real-world study using the French healthcare database. Phys Sportsmed 53(2):159–168 10.1080/00913847.2024.2435256 39618085

[23] Fausett W et al (2019) Physiotherapy treatment and rehabilitation following anterior cruciate ligament injury in New Zealand: Are we doing enough? New Z J Physiotherapy, 47(3)

[24] Ärzteblatt D (2018) Patienten müssen auf Physiotherapietermin lange warten; Available from: https://www.aerzteblatt.de/news/patienten-muessen-auf-physiotherapietermin-lange-warten-aa07c160-b543-4d6c-b827-1238dc497bf2

[25] Andrade R et al (2020) How should clinicians rehabilitate patients after ACL reconstruction? A systematic review of clinical practice guidelines (CPGs) with a focus on quality appraisal (AGREE II). Br J Sports Med 54(9):512–519 10.1136/bjsports-2018-100310 31175108

[26] Del Quiroz A et al (2025) *Arthroskopische Arthrolyse.* Arthroskopie

[27] WIdO (2023) *Heilmittelbericht 2022/2023.*

